# Nanoarchitectonics and Theoretical Evaluation on Electronic Transport Mechanism of Spin-Filtering Devices Based on Bridging Molecules

**DOI:** 10.3390/nano15100759

**Published:** 2025-05-18

**Authors:** Haiyan Wang, Shuaiqi Liu, Chao Wu, Fang Xie, Zhiqiang Fan, Xiaobo Li

**Affiliations:** 1 College of Physics Science and Engineering Technology, Yichun University, Yichun 336000, China; 2 Xiangjiang Laboratory, School of Microelectronics and Physics, Hunan University of Technology and Business, Changsha 410205, China; 3 School of Physics and Electronic Science, Changsha University of Science and Technology, Changsha 410114, China; 4 School of Physics and Optoelectronic Engineering, Hainan University, Haikou 570228, China

**Keywords:** first principle calculation, graphene nanoribbon, spin transport properties, spin-filtering effect, spin-rectifying

## Abstract

By combining density functional theory with the non-equilibrium Green’s function method, we conducted a first-principles investigation of spin-dependent transport properties in a molecular device featuring a dynamic covalent chemical bridge connected to zigzag graphene nanoribbon electrodes. The effects of spin-filtering and spin-rectifying on the *I*–*V* characteristics are revealed and explained for the proposed molecular device. Interestingly, our results demonstrate that all three devices exhibit significant single-spin-filtering behavior in parallel (P) magnetization and dual-spin-filtering effects in antiparallel (AP) configurations, achieving nearly 100% spin-filtering efficiency. At the same time, from the *I*–*V* curves, we find that there is a weak negative differential resistance effect. Moreover, a high rectifying ratio is found for spin-up electron transport in AP magnetization, which is explained by the transmission spectrum and local density of state. The fundamental mechanisms governing these phenomena have been elucidated through a systematic analysis of spin-resolved transmission spectra and spin-polarized electron transport pathways. These results extend the design principles of spin-controlled molecular electronics beyond graphene-based systems, offering a universal strategy for manipulating spin-polarized currents through dynamic covalent interfaces. The nearly ideal spin-filtering efficiency and tunable rectification suggest potential applications in energy-efficient spintronic logic gates and non-volatile memory devices, while the methodology provides a framework for optimizing spin-dependent transport in hybrid organic–inorganic nanoarchitectures. Our findings suggest that such systems are promising candidates for future spintronic applications.

## 1. Introduction

Recent advancements in the miniaturization of conventional electronic devices, coupled with breakthroughs in spintronic technologies [1,2,3], have intensified the demand for novel electronic architectures. Significant progress has been made in understanding spin-dependent transport phenomena through nanostructures and molecular junctions, particularly those exhibiting dual-spin-filtering [4,5,6,7,8], molecular rectification [9,10,11], negative differential resistance (NDR) [12,13,14,15,16], and spin Seebeck effects [17,18,19]. These discoveries underscore the critical importance of fundamental theoretical investigations into electronic transport mechanisms and experimental observations for advancing molecular spintronics. Identifying appropriate molecular materials capable of realizing these phenomena remains a critical research focus. Notable examples include Li et al.’s investigation of spin-polarized transport in p-phenylene vinylene radical-based molecular systems [20] and Xie et al.’s comparative analysis of spin-dependent transport characteristics in biphenyl-bridged zigzag graphene nanoribbon configurations [21], whose results all exhibit excellent spin-filtering and spin rectification effects. Recent developments in modular framework materials, particularly two- and three-dimensional ordered polymers (OPs) such as covalent organic frameworks and metal–organic frameworks, present new opportunities for device engineering. These crystalline porous materials employ dynamic covalent chemistry (DCC) to achieve precise spatial organization of building blocks through geometrically controlled linkages [22,23,24]. Particularly noteworthy is the work of Miao et al. [25], who systematically demonstrated distinct conductance mechanisms in OP-relevant imine-, imidazole-, diazaborole-, and boronate ester-bridging molecules. Despite these advancements, the fundamental spin-dependent electronic transport mechanisms of bridging molecule-based electronic devices remain unexplored. The structures and properties of these bridging molecules play a decisive role in the spin transport performance of molecular devices. However, there are still numerous gaps in our understanding of the role that bridging molecules play in spin transport within molecular devices, and the structure–activity relationship between molecular structures and spin transport performance remains incompletely understood. In light of this, this study conducts in-depth explorations of the spin transport properties of molecular devices based on bridging molecules, aiming to reveal the influence mechanisms of key factors such as intramolecular spin–orbit coupling and molecule–electrode coupling strength on spin transport. The findings of this research will provide theoretical guidance and technical support for the design and fabrication of high-performance spin-based molecular devices.

Breakthroughs in graphene synthesis and the subsequent fabrication of graphene nanoribbons (GNRs) have enabled novel device architectures in spintronics. Pioneering work by Brandbyge et al. [26] established GNR-based two-probe systems as effective platforms for spin injection. Subsequent developments have demonstrated that edge-passivated nanoribbons [27] or magnetically biased electrodes [28] could induce ferromagnetic edge states in zigzag graphene nanoribbons (ZGNRs) [29], thereby enabling spin-valve functionality. The theoretically predicted spin polarization and half-metallicity in ZGNRs [30] have been directly validated at the atomic scale via scanning tunneling microscopy (STM) and spectroscopy (STS) experiments [31]. These results collectively demonstrate that the edge spin polarization in ZGNRs is not merely a theoretical electronic structural property but also an experimentally tunable and robust physical phenomenon, laying a solid foundation for the design of spintronic devices based on ZGNRs. In such configurations, the magnetic alignment between electrodes determines the fundamental operating regime: the P configuration corresponds to identical magnetization orientations, whereas AP alignment occurs when electrode magnetizations exhibit opposing polarization vectors [32].

In this study, we employ first-principles density functional theory (DFT) coupled with the non-equilibrium Green’s function (NEGF) formalism to systematically investigate the spin-polarized electron transport properties of OP-based molecular devices. Three structurally analogous systems, imidazole (CN), diazaborole (BN), and boronate ester (BO) molecular frameworks, were selected to construct nanoscale device architectures. Through comprehensive quantum transport simulations, we elucidated the fundamental charge transport mechanisms and spin-dependent characteristics of these systems. This comparative analysis provides critical theoretical insights into structure–property relationships, thereby establishing a robust foundation for the rational design and implementation of bridging molecule functional devices in next-generation spintronic applications.

## 2. Models and Computational Methods

**Figure 1:**
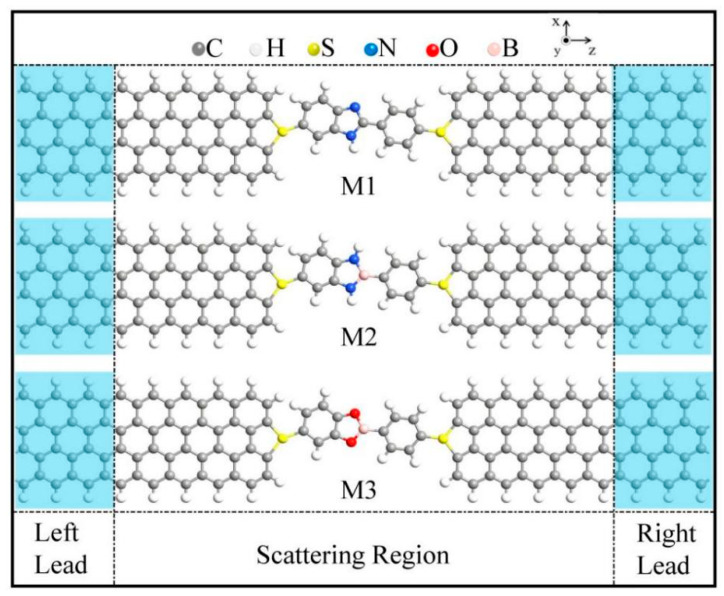
Schematic diagram of the designed molecular junction connected with CN, BN, and BO between the metal ZGNR electrodes, with corresponding device M1–M3, respectively. The left and right electrodes are demarcated by light-blue-shaded frames. Atomic species are color-coded as follows: gray (carbon, C), white (hydrogen, H), red (oxygen, O), yellow (sulfur, S), pink (boron, B), and blue (nitrogen, N) (Note: Color interpretation references are available in the digital version).

Experimental studies [33] have demonstrated that molecular devices with graphene-based electrodes can be constructed and regulated in a controlled manner. In this study, three distinct molecular junctions (denoted M1, M2, and M3) were constructed by anchoring optimized CN, BN, and BO molecules between two 4-zigzag graphene nanoribbon (4-ZGNR) electrodes via sulfur atoms, which are shown in Figure 1, where sulfur atoms were successfully employed to connect π-conjugated molecules to 4-ZGNR electrodes used to keep the spin-dependent transport characteristics. Here, the numerical prefix “4” designates the number of zigzag carbon chains along the direction perpendicular to the nanoribbon axis and defines the width of the ZGNR. Hydrogen passivation was applied to the edges of the ZGNR to eliminate dangling bonds and stabilize the structure. In order to prevent Coulomb interactions between adjacent nanoribbons or periodic structures, a vacuum layer of 20 Å (1 Å = 1 × 10^−10^ m) was set in the x direction. The device architecture comprises three functional regions: a semi-infinite left electrode (L), semi-infinite right electrode (R), and central scattering region containing the molecular interface with buffer layers. The electrodes extend periodically along the transport direction (z-axis), whereas the scattering region incorporates electrode extensions to ensure proper electronic coupling. Electron transport occurs along the principal device axis (z-direction), with the spin-polarization configurations of the two electrodes being tunable between the P (magnetic configuration (1, 1)) and AP (magnetic configuration (1, −1)) states through external magnetic field control.

Furthermore, we systematically investigated the spin-resolved transport characteristics of the proposed molecular junctions using first-principles quantum transport simulations. All computational work, including structural relaxation and spin-polarized transport calculations, was implemented using the Atomistix ToolKit (ATK) platform version 13 [34,35,36], which synergistically integrates DFT [37] with the NEGF [38] formalism. The exchange–correlation interactions were treated within the spin-polarized generalized gradient approximation (SGGA) employing the Perdew–Burke–Ernzerhof (PBE) functional form, and the band structures of the ZGNR exhibit no significant differences when using different computational methods. The structural relaxation of the scattering region was performed using the quasi-Newton algorithm until all atomic forces met a convergence criterion of 0.05 eV/Å (1 eV = 1.6 × 10^−19^ J). Norm-conserving pseudopotentials were utilized to model electron–ion interactions in subsequent electronic structure calculations. For Brillouin zone integration, a Monkhorst–Pack k-point mesh of 1 × 1 × 100 was implemented along the periodic transport direction, with self-consistent field iterations terminated when the total energy difference between consecutive steps dropped below 10⁻⁵ eV [39]. A plane-wave energy cut-off of 150 Ry was applied throughout the calculations. Electron wavefunctions were expanded using a double-zeta polarized (DZP) basis set, with ionic cores represented by Troullier–Martins norm-conserving pseudopotentials. The spin-dependent current–voltage characteristics were subsequently computed through self-consistent implementation of the Landauer–Büttiker formalism [20,40,41,42,43]:

(1) $$I_{\sigma }(V)=\frac{e}{h}\int_{\mu_{L}}^{\mu_{R}}T_{\sigma }(E,V)\left[f_{L}(E-\mu_{L})-f_{R}(E-\mu_{R})\right]dE$$

where σ is the spin state (up/down) of electrons, and e and h represent the electron charge and Planck constant, respectively. $T_{\sigma }$ is the spin-resolved transmission function through the device with the help of the NEGF, which can be calculated by the following equation:

(2) $$T_{\sigma }(E,V)=Tr[\Gamma_{L}(E,V)G_{\sigma }(E,V)\Gamma_{R}(E,V)G_{\sigma }^{†}(E,V)]$$

where Green’s function is defined as $G_{\sigma }(E,V)$ in the central scattering region, and $G_{\sigma }^{†}(E,V)$ is the corresponding complex conjugate. $\Gamma_{L/R}$ is the coupling matrix of the two leads of the molecular junction.

(3) $$f_{L/R}(E,V)=1/[1+e^{(E-\mu_{L/R})/\kappa_{B}T}]$$

is the Fermi–Dirac distribution function of the left and right electrodes, respectively. The chemical potential is $\mu_{L/R}(V)=\mu_{L/R}\pm eV/2$ under external bais. Next, the charge current can be given as

(4) $$I_{P/AP}=I_{P/AP}^{U}+I_{P/AP}^{D}$$

where $I_{P/AP}^{U/D}$ is the transport current calculated from the Landauer–Büttiker formula corresponding to spin-up (SU) and spin-down (SD) electrons. The spin-filtering efficiency (SFE) of the current can be written as follows:

(5) $${\mathrm{SFE}}_{P/AP}=\frac{I_{P/AP}^{U}(E_{F})-I_{P/AP}^{D}(E_{F})}{I_{P/AP}^{U}(E_{F})+I_{P/AP}^{D}(E_{F})}\times 100\%$$

## 3. Results and Discussion

**Figure 2:**
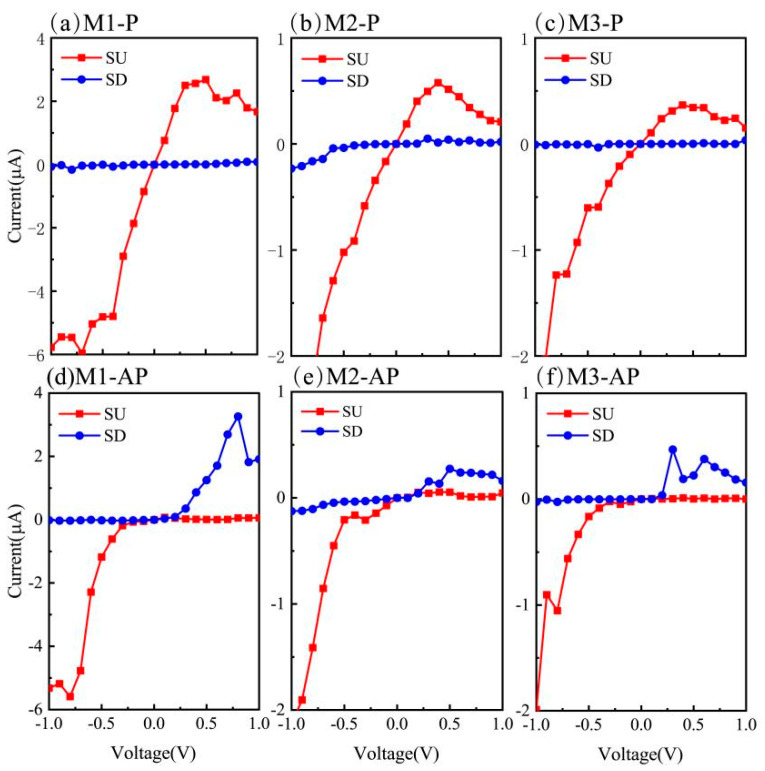
The spin-polarized I-V characteristics of calculated current varying with voltage for M1–M3 devices, where (**a**–**c**) are the currents corresponding to the P magnetic configurations, while (**d**–**f**) correspond to the AP configurations. The red (blue) solid lines represent SU (SD) currents, and the external bias is set from −1 V to 1 V.

The electronic transport characteristics of three structurally analogous molecular devices (M1–M3) were systematically investigated using first-principles quantum transport simulations. Figure 2a–f displays the spin-dependent current–voltage (*I*–*V*) characteristics under the bias from −1 V to 1 V for both P and AP spin configurations. Notably, panels (a)–(c) reveal pronounced spin polarization in the P configuration, where SU currents dominate the entire bias range while the SD currents are negligible. This disparity in spin-dependent conduction channels reveals a remarkable spin-filtering mechanism, suggesting excellent single-spin transport functionality in the P magnetic alignment for all the investigated molecular devices. Meanwhile, a systematic comparison revealed superior SU current magnitudes in device M1 compared to M2 and M3, which suggests distinct spin transport mechanisms arising from subtle structural modifications among the three configurations.

Furthermore, Figure 2a–c show that the SU currents undergo a distinct voltage-dependent evolution; a sharp rise occurs within the low-bias regime (0–0.5 V), followed by a marked decline at higher biases (>0.5 V). This nonmonotonic behavior, characterized by a peak-to-valley transition in the current–voltage profiles, demonstrates the emergence of a pronounced NDR effect across the designed devices under positive bias. The preservation of this NDR signature despite variations in absolute current magnitudes suggests that the underlying quantum transport mechanism governing the peak–valley relationship remains robust against structural modifications in the molecular framework. Such voltage-activated conductance modulation highlights the potential of these molecular systems for tailored electronic functionalities, particularly in nonlinear circuit applications that require tunable NDR characteristics.

Under AP magnetic configurations, as illustrated in Figure 2d–f, the three devices exhibited consistent spin-polarized transport asymmetry. A notable characteristic emerges under negative bias conditions: SU currents are discernible and as high as 6 μA, (1 μA = 1 × 10^−6^ A) which is consistent with the conclusion of an earlier work [25], while SD currents remain near zero. Conversely, under a positive bias, SD currents dominate, with SU contributions rendered negligible. This bidirectional spin selectivity arises from distinct electron localization mechanisms. Specifically, SD electrons experience strong localization under a negative bias, effectively suppressing their participation in transport. Simultaneously, SU carriers are excluded from the conduction pathways under positive bias, owing to interfacial spin-blocking effects. Such voltage-polarity-dependent spin-filtering demonstrates dual-function spin-diode characteristics in the M1–M3 architectures. This bidirectional spin polarization capability indicates that the devices are promising candidates for advanced spintronic applications requiring bias-controlled spin-current modulation.

**Figure 3:**
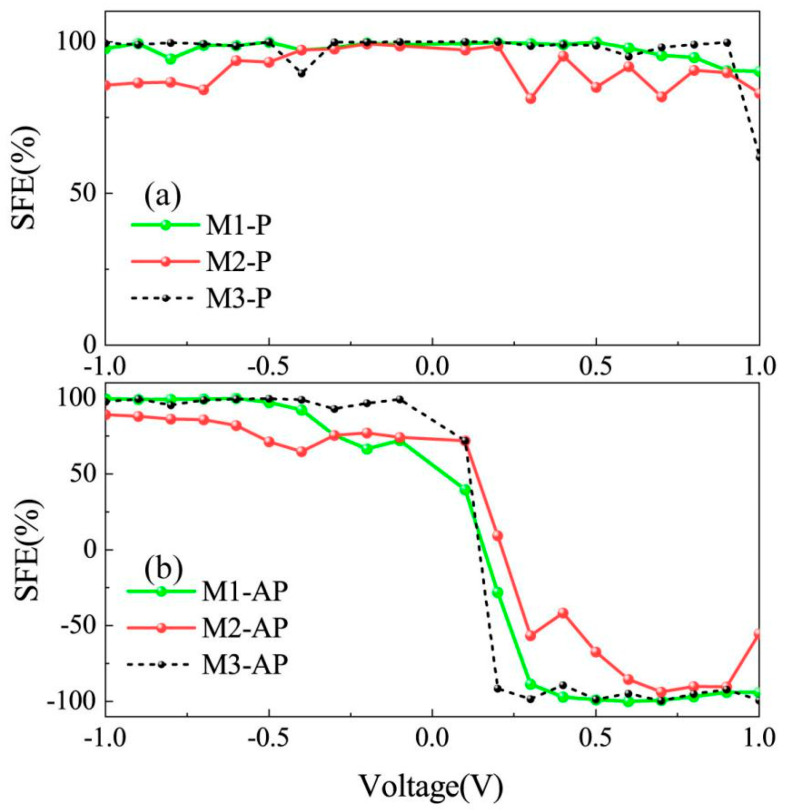
The bias-dependent spin-filtering efficiency (SFE) curves of the devices M1–M3 in the (**a**) P and (**b**) AP spin state, which are described by the green solid, red solid, and black dashed lines, respectively.

To quantitatively evaluate spin-filtering performance, Figure 3 systematically compares the SFE of devices M1–M3 under the P and AP magnetic configurations. As the green solid curve demonstrated in Figure 3a for the M1 device, the black dashed line represents unipolar spin polarization (SFE ≈ 100%) across the entire nonzero bias spectrum in the P configuration, confirming robust single-spin-filtering capabilities. For the M2 device, the red solid curve approaches nearly 100% efficiency within the voltage range of −0.4 V to 0.4 V, while showing significantly reduced performance outside this interval. In contrast, the M3 device demonstrates a black dashed curve that fluctuates around 100% efficiency across a broader voltage window from −1.0 V to 1.0 V, though it exhibits a sharp decline to approximately 60% at the 1 V bias point. All three investigated devices confirm robust single-spin-filtering capabilities, as evidenced by their high-efficiency operational ranges. However, in the AP configuration, there is a striking polarity-dependent inversion of spin selectivity (Figure 3b). As the green solid curve shows, the M1 device remains stabilized near 100% efficiency within the −1.0 V to −0.4 V range, while showing oscillations around −100% efficiency across the 0.3 V to 1.0 V range. In comparison, the M2 device demonstrates weaker spin-filtering efficiency (SFE), with its red solid curve exhibiting pronounced fluctuations, though reaching comparable maximum values. For the M3 device, as shown by the black dashed curve, it maintains highly stable +100% polarization under negative bias conditions, but oscillates around −100% polarization within the positive bias range of 0.2 V to 1.0 V. The oscillations here originate from quantum interference effects [44] inherent to spin-polarized transport in nanoscale junctions. Collectively, all devices display dominant fully SU polarization approaching +100% under negative bias, whereas rapid reversal to −100% spin polarization occurs under positive bias. These systematic observations confirm bidirectional electrical control of spin states through bias polarity switching.

**Figure 4:**
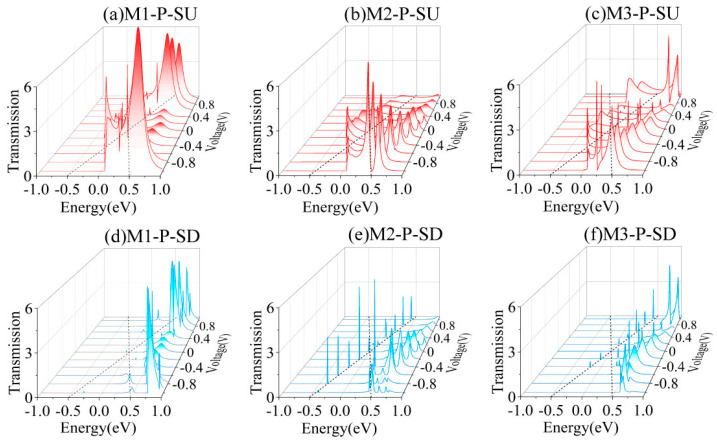
The bias-dependent transmission spectra of M1–M3 in the P spin state. The red and blue solid lines in panels (**a**–**c**) and (**d**–**f**) are the spin-resolved transmission spectra for the SU and SD channels, respectively, with black dashed lines demarcating the energy window defined by the applied bias voltage (EVB).

To clarify the spin-filtering mechanism in devices M1–M3, we analyze the transmission spectra variations under the bias voltages across the −1 V to 1 V bias range in the P magnetic configuration, as presented in Figure 4. According to Landauer theory, the current magnitude through the molecular junction is governed by the integration area of the transmission spectrum within the energy range defined by the applied bias [45,46]. This quantitative relationship establishes that the enhanced spin-polarized current arises from selective transmission peaks aligned within the bias-driven energy window. As illustrated by the red solid curves within the black dashed boundaries in Figure 4a–c, distinct transmission resonances for SU electrons persist across both the positive and negative bias windows, corresponding to the pronounced SU currents observed in Figure 2a–c. In contrast, as shown by the blue solid curves in Figure 4d–f, the SD electrons’ transmission spectra exhibited negligible transmission signals within the operational bias range, with only minor sharp peaks localized near the bias window boundaries. This suppressed SD transmission directly correlates with the nearly zero SD currents measured in Figure 2a–c. The remarkable current disparity between the SU and SD channels demonstrates the nearly ideal spin-filtering efficiency in these molecular devices.

**Figure 5:**
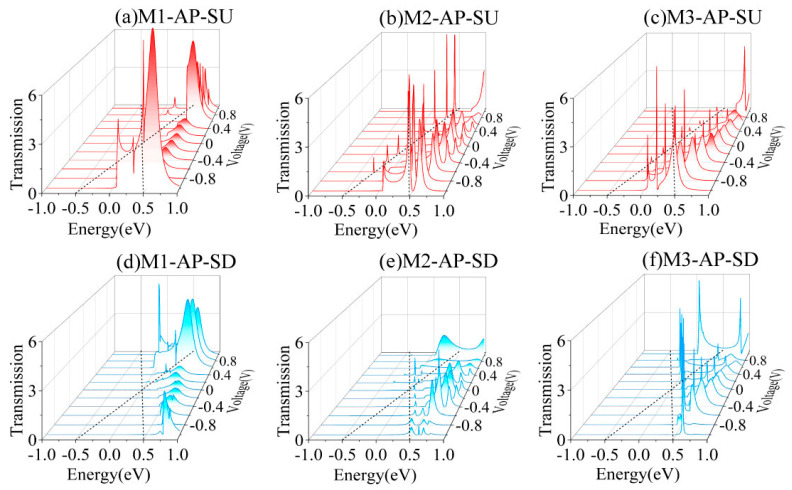
The bias-dependent transmission spectra of M1–M3 in the AP spin state. The red and blue solid lines in panels (**a**–**c**) and (**d**–**f**) are the spin-resolved transmission spectra for the SU and SD channels, respectively, and the EVB is denoted by the black dotted lines.

Figure 5 presents the bias-dependent evolution of the spin-resolved transmission spectra to explain the dual-spin-filtering mechanism in the AP configuration. As shown in Figure 5a–c, under negative bias voltages, prominent transmission peaks emerged exclusively for SU electrons within the energy window defined by the applied bias, but when a positive bias was applied, the SU transmission peaks shifted out of the bias window. Conversely, as shown in Figure 5d–f, for SD electrons, there are no significant transmission features observed under the negative voltage; only when the bias becomes positive do the distinct SD transmission peaks appear within the newly defined energy range. This bias-tunable spin selectivity reveals asymmetric transport behavior: SU electrons dominate conduction under negative bias, whereas SD electrons govern transport under positive bias. These observations directly demonstrate the emergence of a dual-spin-filtering phenomenon, in which the device selectively transmits opposite spin orientations depending on the applied bias polarity, thereby achieving reversible spin polarization controlled by an external voltage.

**Figure 6:**
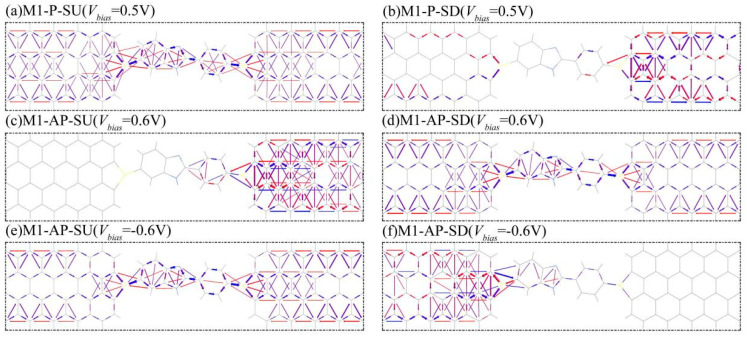
The spin-resolved transmission pathways for device M1. For comparison purposes, (**a**,**b**) shows the transmission pathway of SU/SD electrons at 0.5 V in the P magnetic configuration, while (**c**,**d**) is at 0.6 V with the AP case, and (**e**,**f**) is at −0.6 V with the AP case, respectively. The blue/red arrows indicate electron movement from left/right to right/left electrode, where the value of transmission probability depends on the volume of arrows.

To further systematically investigate the spin-dependent transport mechanisms in M1–M3 systems, we analyzed the electron transport pathways of model M1 at the Fermi level (E_F_ = 0) under various bias conditions in both the P and AP configurations, as shown in Figure 6. Visualization of transmission pathways offers an intuitive method for tracking spin-resolved electron migration patterns across molecular junctions [47]. As demonstrated in Figure 6a,b for M1 in the P configuration under +0.5 V bias, the distinct conduction pathways for SU and SD electrons reveal the spin-filtering mechanism. The directional electron flows are represented by blue arrows (left-to-right electrode transport) and red arrows (right-to-left reverse transport). This spatial distribution of spin-polarized currents directly illustrates how specific spin orientations dominate conduction, depending on the applied voltage polarity, providing visual evidence for the voltage-controlled spin selection process. As shown in Figure 6a, the SU electrons exhibit continuous pathways spanning the entire molecular junction (from the left electrode to the scattering region to the right electrode), indicating unimpeded transport with efficient charge transfer. In contrast, the SD electrons display localized pathways, predominantly confined to the right electrode and scattering region, with negligible electronic distribution in the left electrode, as shown in Figure 6b. This spatial asymmetry in the transmission pathway directly correlates with the observed current disparity: SU electrons dominate conduction owing to their complete transport channels, while SD electrons exhibit severely restricted mobility. Such a pronounced contrast in the pathway connectivity between spin orientations provides direct visual evidence of the spin-filtering phenomenon observed in Figure 2a, where the device preferentially transmits SU electrons under these bias conditions. The suppressed SD current originates from the absence of continuous transmission channels, demonstrating that the electronic structure of the molecular junction selectively enables or blocks specific spin states.

When the bias is equal to 0.6 V in the AP configuration, the SU electrons exhibit localized transmission pathways confined primarily to the right electrode, with negligible activity in the scattering region or left electrode in Figure 6c. Conversely, in Figure 6d, the SD electrons display extended pathways spanning the entire molecular junction, indicating unimpeded transmission through the scattering region. This asymmetric transport behavior results in dominant SD conduction under a positive bias, effectively filtering the SU electrons. However, when reversing the bias to −0.6 V, the spin selectivity inverts. As shown in Figure 6e,f, the SU electrons now demonstrate continuous transmission pathways across the scattering region, whereas the SD electrons show restricted pathways localized near the left electrode, with minimal participation in the scattering region or right electrode. This bias-polarity-dependent reversal of the spin transmission pathway, which means that SU favors a negative bias versus SD favoring a positive bias, directly manifests the dual-spin-filtering mechanism. The observed spatial asymmetry in the pathway distributions correlates with the energy-dependent alignment of spin-resolved molecular orbitals relative to the bias window. Such voltage-controlled switching between spin-selective transport channels enables reversible spin polarization, indicating the capability of the system for bidirectional spin discrimination under opposite bias voltages. This mechanism provides a microscopic explanation for the dual-spin-filtering behavior observed in Figure 2d.

**Figure 7:**
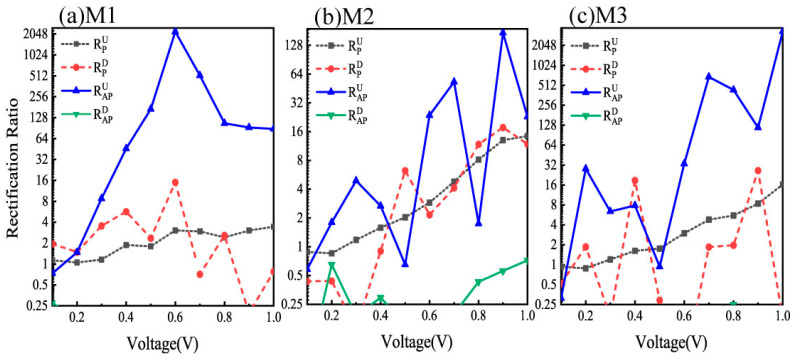
The spin-resolved rectification ratio as a function of bias voltage for device models M1–M3 (**a**–**c**) with P/AP magnetizations configuration, and the rectification ratios below 0.25 are ignored.

To evaluate the rectification performance comprehensively, Figure 7 presents the spin-resolved rectification ratios (RRs) as functions of the bias voltage for devices M1–M3 ((a)–(c)) with different configurations. The RRs were derived from the current–voltage (*I*–*V*) characteristics in Figure 2 using the following conventional formula: $R_{P/AP}^{U/D}(V)=\left|I_{P/AP}^{U/D}(-V)\right|/I_{P/AP}^{U/D}(V)$, where U/D denotes SU/SD channels in P and AP configurations. As shown by the blue solid curve in Figure 7a for device M1, the SU rectification ratio $R_{AP}^{U}$ exhibits a prominent peak value of 2209 at 0.6 V bias, whereas the other configurations demonstrate relatively flat responses with significantly lower values across the entire bias range. In contrast, Figure 7b reveals that device M2 generally exhibits inferior rectification performance, except for a moderate $R_{AP}^{U}$ peak of 172 at 0.9 V bias. This marked performance degradation compared to M1 suggests that substituting atom C with B substantially diminishes the rectification capability of the system. Notably, device M3 in Figure 7c demonstrates exceptional rectification enhancement, particularly for $R_{AP}^{U}$, which reaches a remarkable peak value of 3310 at 1.0 V bias, the highest observed among all configurations. A comparative analysis of the three devices revealed significantly stronger rectification performance in M1 and M3 than in M2. The predominant enhancement in SU RRs in the AP configuration indicates that the transmission asymmetry between positive and negative biases is most pronounced for SU electrons in these optimized device geometries.

**Figure 8:**
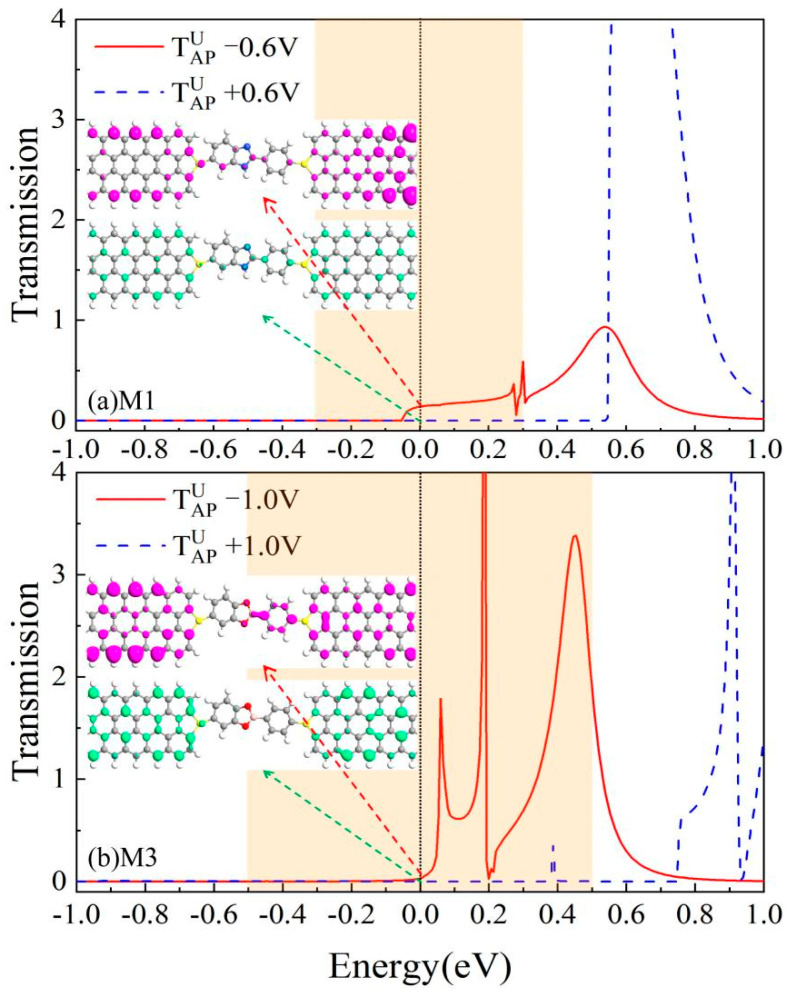
The spin transmission spectrum as a function of the energy for (**a**) M1 at ±0.6 bias and (**b**) M3 at ±1.0 bias in the AP state. The corresponding local density of state (LDOS) at the Fermi level is displayed in panels (**a**,**b**).

The rectification characteristics of electronic devices originate from spatial asymmetry in electron localization [48], which can be further corroborated through bias-dependent transmission spectra and LDOS analysis. Figure 8 presents the spin-polarized transmission spectra in the AP spin configuration for (a) M1 and (b) M3 systems, where red solid and blue dashed curves correspond to negative and positive bias conditions, respectively. We can see that there are wide and distinct transport peaks in the window under negative voltage bias, while it is near zero under positive bias. Therefore, the greater integrated areas within the bias window under negative bias compared to positive bias configurations, as well as the larger value of the spin-polarized current under negative bias than that under positive bias, result in the rectification effect for M1 [see $I_{AP}^{U}$ under bias ±0.6 V shown in Figure 2d] and for M3 [see $I_{AP}^{U}$ under bias ±1.0 V shown in Figure 2f]. The accompanying LDOS plots at the E_F_ reveal that for M1, the SU electrons demonstrate delocalized wavefunctions across the entire molecular framework under a negative bias, whereas the electrons show extremely sparse atomic-scale localization across the left electrode, right electrode, and central scattering region under a positive bias. For M3, under negative bias, the SU electrons exhibit spatially extended wavefunctions across the entire molecular junction, whereas under positive bias, they are strongly confined to the terminal regions with nearly zero electronic density on the central units. This implies voltage-polarity-dependent rectification behavior.

## 4. Summary and Conclusions

In this study, we systematically investigated the spin-dependent transport properties of ZGNR-based devices bridged with CN, BN, and BO molecular junctions using DFT combined with the NEGF method. Remarkably, our findings reveal that all three devices demonstrate near-perfect single-spin-filtering effects in the P spin configuration and dual-spin-filtering effects in the AP spin configuration. The spin polarization ratios demonstrated are nearly 100% across the entire bias range, indicating that the devices produce spin-polarized currents with relatively high spin polarization efficiency and emphasizing their potential as high-efficiency spin filters. Meanwhile, all systems exhibited a weak NDR effect under positive bias conditions. Furthermore, the inherent structural asymmetry of the bridging molecule units in the central scattering region leads to pronounced spin rectification behavior. The rectification ratio of the SU electrons in the AP configuration is high as 2209 for M1 and 3310 for M3, which is reasonably explained by the transport spectrum of the SU electrons under positive/negative bias and the LDOS at the E_F_ in the AP configuration. These findings establish a universal design paradigm for achieving dual-spin-filtering and high rectification in asymmetric molecular junctions, transcending the specific materials studied here. The synergy between structural asymmetry and spin-polarized interfacial coupling offers a generalizable route to engineering multifunctional spintronic components, such as spin diodes and reconfigurable logic units, across diverse molecular electrode architectures. Moreover, the observed weak NDR effect suggests potential extensions to low-power memory devices or neuromorphic computing platforms. These results provide critical theoretical insights for designing multifunctional spintronic devices that integrate ZGNR electrodes with bridging molecules, highlighting their potential for high-performance spintronic applications.

## Data Availability

The data presented in this study are available on request from the corresponding authors.
